# An Atypical Presentation of a Pyogenic Liver Abscess Caused by Fusobacterium nucleatum Post-ruptured Appendix

**DOI:** 10.7759/cureus.42895

**Published:** 2023-08-03

**Authors:** Hinal Rathi, Faraeha Fatima, Taimoor Hassan, Kiely Schultz

**Affiliations:** 1 Internal Medicine, Arkansas College of Osteopathic Medicine, Fort Smith, USA; 2 Internal Medicine, Parkview Medical Center Internal Medicine Residency Program, Pueblo, USA

**Keywords:** fusobacterium treatment, appendicitis, fusobacterium liver abscess, fusobacterium nucelatum, pyogenic abscess

## Abstract

A rare formation of a pyogenic liver abscess can be found in patients with a ruptured appendix. Here, we discuss a 49-year-old male with a past medical history significant for drug-induced pancreatitis from metformin, type II diabetes, obesity, and hypertension who presented with right upper quadrant pain. He was diagnosed with a 12.6 cm pyogenic liver abscess. Upon further chart review, the patient had a laparoscopic appendectomy done nine months ago, with the findings of a ruptured appendix. The liver abscess cultures grew*Fusobacterium nucleatum* - a common culprit of acute appendicitis reported in several case studies and clinical trials. This case report highlights the importance of including appendectomy as an essential part of history-taking and diagnostic differential for pyogenic liver abscesses.

## Introduction

Liver abscesses are usually two types - pyogenic and amebic: relatively rare with an occurrence of 4.1 per 100,000 cases in the United States [1]. Pyogenic liver abscess is the most common and caused by bacterial infections, such as *Escherichia coli, Enterococcus, Staphylococcus, Streptococcus*, and anaerobes [1-2]. Such liver abscesses are formed due to bowel content leakage, which travels to the liver through the portal vein and causes peritonitis. As such, an anaerobe is *Fusobacterium nucleatum*, a Gram-negative fastidious anaerobe, most commonly found in the oral cavity [3]. It invades through epithelial and endothelial cells. It is an adhesive bacterium that uses the *Fusobacterium *adhesin A (FadA) virulence factor for colonization and dissemination [4]. The inflammatory response of *Fusobacterium nucleatum* is increased through the retinoic acid-inducible gene I pathway, making it easier for the bacteria to invade and spread to other parts of the body through colonization [4]. More recently, in several case studies and clinical trials, *Fusobacterium nucleatum* has been found in pathological cultures of appendicitis [3,5]. In another case study, the formation of a pyogenic liver abscess was linked to acute appendicitis [2]. This establishes a potential link between acute appendicitis and the development of a subsequent pyogenic liver abscess. Here, we report a rare case of pyogenic liver abscess growing *Fusobacterium nucleatum* secondary to a ruptured appendix with peritonitis.

## Case presentation

A 49-year-old male, with a significant past medical history of drug-induced pancreatitis from metformin, type II diabetes, obesity (body mass index of 41.1), and hypertension, presented to the Emergency Department (ED) with complaints of fever, cough, shortness of breath, and right upper quadrant (RUQ) pain. His generalized symptoms started six days ago with the abdominal pain starting in the past two days. He had another ED visit for his generalized symptoms six days before his current admission where he was prescribed doxycycline for suspicion of upper respiratory tract infection. His condition did not improve, and he presented back in the ED now, in addition to abdominal pain. Past surgical history was significant for appendectomy and bilateral foot fracture surgery. Social history is significant for no alcohol and no tobacco use does recreational marijuana, last use was six months prior to this admission. Home medications include 5 mg of glipizide daily, 15 units of Lantus at night, 40 mg of atorvastatin, and 10 mg of lisinopril daily. The patient reports compliance with his home medications.

**Figure 1:**
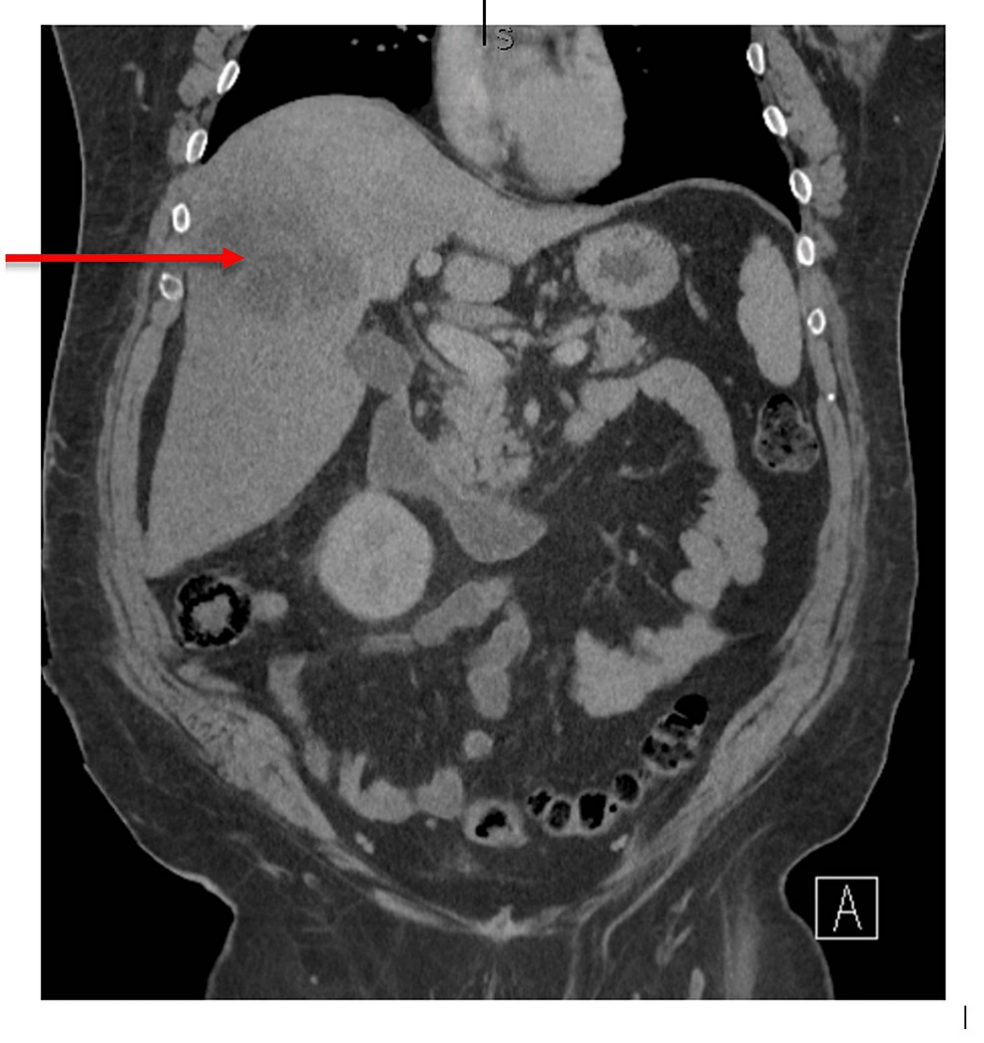
Computer tomography (CT) abdomen and pelvis without contrast findings: There is a large ill-defined hypoattenuating lesion or developing fluid collection within the right lobe of the liver, a lesion measuring collection within the right lobe of the liver, and a lesion measuring roughly 12.6 x 9.8 cm. The more central area of hypoattenuation measures slightly smaller, measuring up to 8.8 x 8.6 cm. There is decreased hepatic attenuation.

In the ED, a sepsis workup was initiated as the patient was tachycardic, febrile, and tachypneic and had an elevated white blood cell count of 16.64 x 10^9^/L. He was given a one-time dose of 2 g of intravenous (IV) ceftriaxone, 500 mg of IV Flagyl, and 2,259 ml bolus of normal saline. Blood cultures were also taken. He had a computer tomography (CT) scan of his abdomen and pelvis without contrast, with findings of a 12.6 cm hypoattenuating mass and/or fluid collection within the right lobe of the liver (Figure 1). Differential considerations include a large necrotic mass/malignancy. Interventional radiology was consulted for drainage. A liver abscess was found on drainage and was sent for cultures. The gram stain showed purulent inflammation, consistent with hepatic abscess; the special stain was negative for amoeba. Anaerobic cultures showed *Fusobacterium nucleatum* growth with sensitivity results showing pansensitive. The patient has continued on the empiric antibiotic regimen of 1 g of IV ceftriaxone every 24 hours and 500 mg of IV Flagyl every eight hours. The infectious disease department was consulted, and they recommended 1 g of amoxicillin every eight hours for the next 30 days after discharge.

A few months later, a repeat CT scan done outpatient showed an area of hypoattenuation in the right lobe significantly reduced from previous CT post drainage and antibiotics. However, while the patient was admitted to find out the possible source of his pyogenic liver abscess on further chart review, it was found that the patient’s laparoscopic appendectomy was done nine months ago. He had a one-day hospitalization after his appendectomy and was discharged on amoxicillin-clavulanate for a duration of three days. During his appendectomy, the patient was found to have a perforated appendix. The surgical pathology report confirmed acute appendicitis with peritonitis. It is nine months after the appendectomy the patient presented with RUQ pain.

## Discussion

We report a case of a 49-year-old male who developed a secondary pyogenic liver abscess growing *Fusobacterium nucleatum* nine months after having an appendectomy, with findings of a ruptured appendix. As mentioned before, several studies have identified this bacterium as one of the causes of appendicitis [2-3,5]. In the United States, pyogenic abscess caused by* Klebsiella pneumoniae* is becoming more common [6-7]. In one case study, it was reported that a pyogenic liver abscess developed following an appendectomy caused by *Streptococcus milleri* [2]. Hence, it is uncommon and rare to come across a case where *Fusobacterium nucleatum* is the causative bacterium. It is also interesting to note the formation of a pyogenic liver abscess occurring nine months after a patient’s appendectomy. Usually, it takes a few weeks for a pyogenic liver abscess to develop [1]. In addition, despite the patient receiving antibiotics after their appendectomy, which should have lowered the risk of a pyogenic liver abscess, it was still unexpected for one to develop.

Furthermore, a similar case report of a liver abscess caused by *Fusobacterium nucleatum* resulted in an appendectomy due to an inflammatory scar extending from the sigmoid rectum to the appendix [8]. In our case, acute appendicitis was first treated with an appendectomy, but the subsequent growth of a liver abscess required further intervention. Studies have also shown that liver abscesses caused by *Fusobacterium nucleatum* can also develop due to periodontitis, Lemierre's syndrome, and celiac disease [9-11]. This case is one of the earliest reports of such an abscess following an appendectomy due to a ruptured appendix.

To highlight pyogenic liver abscesses, males predominately develop these abscesses over females; the mean age group is 62.4 years in the United States [1]. Patients with a liver transplant history, history of malignancy, alcoholism, autoimmune processes such as rheumatoid arthritis or systemic lupus erythematosus, and diabetes mellitus have an increased risk of developing this disease process. Clinical presentations may vary [1,8]. Most patients will present with fever, chills, abdominal pain, or fatigue [1]. The initial evaluation should include a complete blood count panel, a comprehensive metabolic panel, and imaging studies, such as CT abdomen and pelvis without contrast. Treatment with empiric antibiotics should be started if a liver abscess is suspected with the plan to modify antibiotics later in the hospital course. For empiric therapy, it is important to cover streptococci, Gram-negative bacilli, and anaerobes. Management includes abscess drainage, with radiological-guided percutaneous drainage replacing surgery [12]. Surgical intervention is reserved for patients unsuitable for percutaneous drainage, such as patients with multiple abscesses.

Overall prognosis is good for the standard treatment with one population-based study showing a mortality rate of 0.22 per 100,000 population [13]. Mortality is higher in patients with community-acquired pyogenic liver abscesses, liver transplantation, and in older male populations [13]. Other poor prognostic factors include elevated white count, BUN, serum creatinine, total bilirubin, low levels of serum albumin, low serum hemoglobin, and liver abscess of biliary origin [1,12]. In conclusion, pyogenic liver abscess due to acute appendicitis is rare but should be on the index of suspicion when dealing with patients who have the above-stated symptoms and risk factors. History and physical examination are important to find recent or current appendicitis.

## Conclusions

This case emphasizes the significance of a comprehensive surgical history in RUQ pain and has a high index of suspicion for liver abscesses especially in those with multiple health conditions. Such patients have a high chance of experiencing septic shock and increased mortality due to the delayed detection of the abscesses. Additionally, it is important to consider *Fusobacterium nucleatum* as a potential cause of pyogenic liver abscess resulting from a ruptured appendix.
